# The application of near infrared spectroscopy in nutritional intervention studies

**DOI:** 10.3389/fnhum.2013.00473

**Published:** 2013-08-13

**Authors:** Philippa A. Jackson, David O. Kennedy

**Affiliations:** Faculty of Health and Life Sciences, Brain, Performance and Nutrition Research Centre, Northumbria UniversityNewcastle upon Tyne, UK

**Keywords:** NIRS, nutrition, cognition, neuroimaging, intervention studies

## Abstract

Functional near infrared spectroscopy (NIRS) is a non-invasive optical imaging technique used to monitor cerebral blood flow (CBF) and by proxy neuronal activation. The use of NIRS in nutritional intervention studies is a relatively novel application of this technique, with only a small, but growing, number of trials published to date. These trials—in which the effects on CBF following administration of dietary components such as caffeine, polyphenols and omega-3 polyunsaturated fatty acids are assessed—have successfully demonstrated NIRS as a sensitive measure of change in hemodynamic response during cognitive tasks in both acute and chronic treatment intervention paradigms. The existent research in this area has been limited by the constraints of the technique itself however advancements in the measurement technology, paired with studies endeavoring increased sophistication in number and locations of channels over the head should render the use of NIRS in nutritional interventions particularly valuable in advancing our understanding of the effects of nutrients and dietary components on the brain.

## Introduction

Near infrared spectroscopy (NIRS) is a non-invasive optical imaging technique used to monitor tissue oxygen status. In the brain, NIRS can be used to examine cerebral blood flow (CBF) and the local hemodynamic response during neural activity. The technique was originally described by Jobsis (1977) although it is only in the last two decades that NIRS has been successfully used to assess *in vivo* functional activation, following the first demonstrations by Tamura and colleagues in Japan (Hoshi and Tamura, 1993a,b). During this time the technique has been widely applied in clinical settings. However, only recently has the technology been employed in the field of nutritional neuroscience. Given that this technique has the advantage of being low cost and user friendly in comparison to other neuroimaging methodologies it is ideally suited for use in this area.

This review will cover the basics of NIRS examinations, the validity of NIRS in the assessment of brain function, the utility of NIRS in nutrition intervention studies and the challenges and future directions of NIRS in the field.

## Principles of near infrared spectroscopy and NIRS methods

Light easily passes through biological tissue in the near infrared spectrum (650–1000 nm), allowing for easy and non-invasive illumination of brain tissue via transmission of light through the intact scalp, skull and subarachnoid space to the top layers of the cortex. As oxygenated hemoglobin (oxy-Hb) and deoxygenated hemoglobin (deoxy-Hb) are light absorbing pigments, or chromophores, which absorb light at slightly different wavelengths (800–940 nm and 600–750 nm, respectively), it is possible to measure their concentration by quantifying the amount of light absorbed in these wavelengths during the transit of light through the brain tissue (Chance et al., 1988; Obrig and Villringer, 2003; Okui and Okada, 2005). In the brain, cerebral vessels, glia and neurons work intimately to ensure the constant supply of blood borne energy substrates (i.e., oxygen and glucose) to active areas via a complex series of mechanisms controlled by numerous mediators (reviewed in Girouard and Iadecola, 2006). NIRS, like fMRI, exploits the so-called “neurovascular coupling” of neuronal activity and blood-flow to indirectly assess neuronal activity by measuring changes in oxy-Hb and deoxy-Hb. These chromophores provide proxy measures of blood flow and the consumption of oxygen by neuronal cells in the interrogated tissue. NIRS outcomes can therefore be taken to infer local neural activation or, alternatively, can be interpreted more directly as simple changes in blood flow/volume in underlying cortical tissue. The latter is particularly pertinent as both CBF and the magnitude of the hemodynamic response to neural activity decrease with normal ageing and in neurological disease (Girouard and Iadecola, 2006). A typical response during local neural activity will be seen as an increase in oxy-Hb as blood flow increases in the active tissue, coupled with a concurrent decrease in deoxy-Hb as its concentration reduces in the face of the sudden influx of oxy-Hb (Obrig and Villringer, 2003). Delivery of oxygenated blood to active areas typically exceeds local oxygen utilization and therefore increased oxy-Hb is observed throughout the period of sustained activation. The total concentration of hemoglobin (total Hb) is the sum of oxy-Hb and deoxy-Hb and typically follows the same pattern as oxy-Hb given the aforementioned “overshoot” in cerebral oxygenation (Obrig et al., 1997). In some instances it is possible to observe a transient initial “dip” in oxy-Hb as pre-existing oxygen is extracted by the active neurons prior to the influx of oxygenated blood to the area (Buxton, 2001). In general, changes in oxy-Hb and deoxy-Hb are therefore related to changes in cerebral metabolic rates (Tamura et al., 1997) and total Hb is closely related to CBF/cerebral blood volume (CBV) (Steinbrink et al., 2006).

In terms of practical apparatus, light is introduced to the brain via laser emitting diodes that are placed directly onto the scalp and held in place via a headband. Light then passes from this source to photodetectors, or optodes, which are placed at a set distance away (usually >3 cm), passing through the scalp, skull and underlying brain tissue in a predictable banana-shaped pattern (Okada and Delpy, 2003; Mansouri et al., 2010). Brain tissue is assumed to be a homogenous scattering medium, therefore any attenuation of light in each chromophore specific wavelength can be assumed to be due to absorption by oxy-Hb and deoxy-Hb (Fallgatter and Strik, 1997). A modified Beer-Lambert law incorporating the optical differential path-length factor is used to calculate changes in concentrations of oxy-, deoxy- and total Hb in micro molar units (Pellicer and Del Carmen Bravo, 2011).

A number of different types of NIRS systems exist, each with their own set of advantages and disadvantages, although all systems share common attributes that make it a particularly attractive alternative to fMRI, widely perceived to be the current “gold standard” in neuroimaging. All NIRS systems benefit from high temporal resolution due to the fact that they can sample at 50 Hz or more. In addition, due to the fact that NIRS monitors both oxy- and deoxy-Hb in tandem (as opposed to just the comparative concentrations of oxy-Hb and deoxy-Hb with respect to each other, as in the fMRI blood oxygen level dependent [BOLD] signal), the pattern and time course of hemodynamic response can therefore be measured with a high degree of accuracy. Attachment of the diodes to the head using a simple and relatively comfortable headband also allows for extended recording over several hours. The portability of NIRS systems even accommodates accurate measurement while participants move around, supporting advances in the study of cortical control of gait, for example, but allowing for more ecologically valid paradigms overall (Wang et al., 2008; Kurz et al., 2012). On the other hand, with NIRS spatial resolution is limited to approximately 2 cm as the source and photodetectors must be spaced at a sufficient distance to allow sensitive measurement of tissue absorption (Fukui et al., 2003).

NIRS systems vary in complexity ranging from simple one or two channels to arrays of several dozen that cover the whole head. Several different systems are also available: “time domain” systems emit short bursts of photons, with the temporal distribution providing information about tissue absorption and scattering; “frequency domain” systems emit amplitude-modulated light and record amplitude decay and phase shift; and the most common measurement technique is a “continuous wave” (CW) system that emits light continuously at constant amplitudes, and only the amplitude decay is measured (Strangman et al., 2002). Frequency and time domain systems offer deeper cortical illumination than CW systems—which only illuminate the top 2–3 mm of the cortex (Chance et al., 1988)—as well as the ability to produce absolute values for changes in concentrations of the chromophores. However, compared to CW systems, they are bulkier and much more expensive, rendering these systems less accessible and applicable to a less diverse range of paradigms (Ferrari and Quaresima, 2012). Recent “quantitative” systems have similar advantages in terms of cost and practical considerations as earlier CW systems but more importantly resolve the measurement issue by collecting light at several increasing distances from the light source, allowing the exact calculation of the path length and thereby the absolute quantity of hemoglobin in underlying tissue.

## Validity of NIRS examinations

Despite the limitations of the technique, NIRS has been successfully used to image activation in the frontal cortex (e.g., Fallgatter and Strik, 1997, 1998) and other areas of the brain including the temporal, visual and parietal cortices (e.g., Jasdzewski et al., 2003; Schecklmann et al., 2008a). NIRS has also been particularly valuable as neurovascular assessment tool and for the identification of brain injury in all age groups from neonates (Grant et al., 2009; Lin et al., 2013) to adults (Cohn, 2007; La Monaca et al., 2010; Len and Neary, 2011), and for the evaluation of cognitive function across the lifespan as well as in abnormal functioning. Advances in the field of neonatal and infant cortical functioning have been made possible with NIRS, as the technique is truly non-invasive and can accommodate a certain degree of movement. Evaluations of infant hemodynamic response following simple visual and auditory stimuli have been followed up more recently by studies that have used whole head multi-channel arrays to characterize activation during much more complex tasks including object, face and motion processing and social communication (reviewed in Lloyd-Fox et al., 2010). NIRS has also been used to investigate age-related changes in cerebral oxygenation elicited by sensory stimuli or during cognitive tasks. Reduced hemodynamic response has been generally observed in older, compared to younger participants, as well as subtle differences in cortical control across the cohorts (Schroeter et al., 2003; Kameyama et al., 2004; Safonova et al., 2004; Harada et al., 2007; Kim et al., 2011).

NIRS has also proven to be a sensitive research technique in patients with neurodevelopmental or neurological conditions. For example, reduced hemodynamic response has been demonstrated in the prefrontal cortex in schizophrenia (Shimodera et al., 2012; Taniguchi et al., 2012) as well as in children and adults with Attention Deficit Hyperactivity Disorder (ADHD) (Schecklmann et al., 2008b; Xiao et al., 2012). Prefrontal reductions in tissue oxygenation have also been observed in mild cognitive impairment and dementia (Arai et al., 2006; Herrmann et al., 2008) as well as similar reductions in parietal regions (Zeller et al., 2010). Given these demonstrations, consideration has been given to the possibility of using NIRS as a tool in early disease detection or for supporting accurate clinical diagnosis. For example, one research group in Japan have been able to characterize hemodynamic response to a verbal fluency task in different disease states (Fukuda and Mikuni, 2012). In a similar vein Takahashi et al. (2010) were able to identify differences in prefrontal hemodynamic response to a response inhibition task in responders and non-responders to treatment with methylphenidate (used to treat the symptoms of ADHD), raising the possibility of being able to use NIRS to tackle the issue of over prescription of this drug in children.

Although the number of publications to date is, as yet, still limited, NIRS has also been successfully applied in pharmacological studies where the CBF effects of a wide range of pharmaceutical interventions have been assessed across the lifespan. For example, the use of NIRS in this field has enabled easy and accurate monitoring of neonatal cerebral oxygenation following administration of routine drugs including anesthetic (Vanderhaegen et al., 2010) and non-steroidal anti-inflammatories (Garner et al., 2012), both of which have been shown to reduce cerebral oxygenation. Reductions in the prefrontal CBF response to tasks have been observed following methylphenidate in children with ADHD (Weber et al., 2007), and also in normally developing children following anti-histamines (Tsujii et al., 2009, 2010). Interestingly, Tsujii et al. (2010) were able show the difference in cerebral oxygenation following sedating and non-sedating anti-histamine variants, with reductions in CBF only being observed for the sedating variety. In adults, NIRS has been used to show decreased frontal lobe oxygenation following sumatriptan administration in migraine sufferers (Watanabe et al., 2011) and also following the vasopressor agent phenylephrine, wherein the technique has been instrumental in providing evidence in support of sympathetic regulation of cerebral circulation (Brassard et al., 2010; Ogoh et al., 2011). Conversely, increased frontal CBF has also been demonstrated following the vasodilator vinpocetine in stroke patients (Bonoczk et al., 2002).

## The application of NIRS in nutrition intervention studies

NIRS has been shown to be a feasible method with which to measure hemodynamic response and CBF in the superficial cortex and as a sensitive measure of change in cerebral oxygenation following pharmacological agents. With the growing interest in the contribution of nutrition to cognitive health across the lifespan, the application of NIRS in the field of nutritional neuroscience is a valid and natural extension of the technique. To date, however, only a small number of studies have assessed the cerebral hemodynamic effects of dietary components, and the majority of these have only collected data from the prefrontal cortex. In terms of acute administration studies, two studies have demonstrated the vasoconstricting properties of caffeine. An early study presented by Higashi et al. (2004) found reductions in CBV during the post-treatment rest period compared to the control (no treatment) rest period but found no difference between hemodynamic response to cognitive tasks between the treatment and control test periods, despite performance on the tasks being improved following caffeine. However more recently, Kennedy and Haskell (2011) reported a prefrontal decrease in total Hb during task performance following an ecologically valid dose of caffeine (75 mg). The latter study benefitted from a double-blind, placebo-controlled design, with continuous monitoring during the absorption and testing periods as well as increased statistical power resulting from monitoring NIRS outcomes during multiple completions of the same tasks. This approach has been applied by the same research group to acute investigations of the effects of polyphenols. Examples include demonstrations of a dose-related increase in total Hb and deoxy-Hb during cognitive tasks following 250 mg and 500 mg of resveratrol (Kennedy et al., 2010) and a decrease in total Hb following a single dose of epigallocatechin gallate, the principal polyphenol found in green tea (Wightman et al., 2012). The only study to apply a multi-channel, multi-site approach assessed the immune system marker and CBF effects of acute administration of soybean peptide using a frequency wave NIRS system (Yimit et al., 2012). The authors of this placebo-controlled crossover study reported immediate (30 s) post-treatment (resting) increases in frequency amplitude across several areas of the brain, including the premotor cortex, primary motor cortex and dorsolateral prefrontal cortex.

NIRS has also been successfully applied to chronic intervention studies. Jackson and colleagues assessed the effects of 12 weeks’ administration with fish oil in healthy young adults (18–35 years) on prefrontal oxygenation in two separate double-blind placebo-controlled parallel group studies. The first of these revealed that administration with docosahexaenoic acid (DHA)-rich fish oil, but not eicosapentaenoic acid-rich fish oil, was associated with task-related increases in oxy-Hb and total Hb (Jackson et al., 2012b), whilst the second, larger study was able to demonstrate a dose-related effect of DHA-rich fish oil on the same parameters (Jackson et al., 2012a). Watanabe et al. (2002) also examined cerebral oxygenation in healthy young adults during a mental arithmetic task before and after five days’ supplementation with creatine. Although these authors reported that creatine led to decreased oxy-Hb and increased deoxy-Hb in comparison with the pre-treatment hemodynamic response to the task, they did not conduct any comparisons between the placebo and treatment groups.

## Methodological limitations and future recommendations

The above collection of studies provide strong support in favor of NIRS as a sensitive technique for monitoring changes in hemodynamic response/CBF following the administration of nutrients or dietary components. As yet, however, the application of NIRS in a nutritional neuroscience setting has been limited by the shortcomings of the methodology and also by intrinsic difficulties in applying a relatively novel technique in a field that has only really flourished in the past few decades itself. For example, Tsujii et al. (2011), in a placebo-controlled counterbalanced crossover trial, set out to assess the neural correlates of the effect of alcohol on response inhibition, a process that has previously been associated with activity in the inferior frontal cortex (IFC). These authors used NIRS to assess hemodynamic response in the IFC during a Go/No-Go task prior to and after consuming alcohol and were able to demonstrate for the first time the contribution of the right IFC in inhibitory control of pre-potent responses following alcohol. In addition, the authors reported a negative correlation between increased concentrations of oxy-Hb in the right IFC and false alarms on the task. This type of focused and hypothesis-driven research is simply much more difficult to conduct when the *in vivo* physiological and cognitive effects of the nutritional interventions in question are not as well established, certainly in comparison to pharmacological agents. In addition, given that complications arise from the placement of the optodes on parts of the head that are covered in hair, the research to date has tended to concentrate on examining CBF and hemodynamic responses in the prefrontal region, measured through the hair free forehead. This shortcoming may contribute to the fact that, hitherto, the nutritional intervention studies listed above have failed to find a direct relationship between the observed changes in hemodynamic response/CBF and concomitant significant changes in cognitive function. Furthermore, the depth penetration issues of the NIRS signal—at least with the most prevalent CW NIRS systems—places a further limitation on the areas of the brain that can be assessed. On a positive note, the advent of newly developed quantitative NIRS systems will address the current issue of not being able to report absolute concentration changes in the chromophores, which again has placed limitations on nutritional intervention study designs thus far. With the development of more complex protocols incorporating multi-channel, multi-site monitoring along with the continued investment in the development of the NIRS systems, it is hoped that these challenges will be overcome in future studies.

One last point to note is the current lack of standardized statistical analysis approaches for NIRS assessment, similar to those applied in more well-established neuroimaging techniques. At present there are no universally adopted methods for the data analysis used in the production of topographical maps, or even agreement about determining the statistical significance of hemodynamic changes (Elwell and Cooper, 2011). Further, papers do not always present both oxy- and deoxy-Hb, despite evidence that that they should be equally valued as markers of cerebral oxygenation, and should therefore be reported together as standard.

Overall, NIRS as a neuroimaging technique offers several advantages in nutritional intervention studies over more commonly used methods. Given the increasing demand for functional activation data as a secondary endpoint the fact that NIRS is low cost and practically ready to use “off the shelf” offers a real opportunity for enhancing nutritional intervention studies. However more hypothesis-driven research in the area is a necessity, and the fact that NIRS can easily be combined with other imaging techniques may be one way in which to address this issue. Moreover, the development of quantitative NIRS systems brings with it the real possibility of conducting the type of long-term research that can be informative about the modulating effects of nutrients on the brain across the lifespan.

## Conflict of interest statement

The authors declare that the research was conducted in the absence of any commercial or financial relationships that could be construed as a potential conflict of interest.
